# Self-medication among Medical Students and Staffs of a Tertiary Care Centre during COVID-19 Pandemic: A Descriptive Cross-sectional Study

**DOI:** 10.31729/jnma.7247

**Published:** 2022-01-31

**Authors:** Anna Acharya, Marina Vaidya Shrestha, Dimindra Karki

**Affiliations:** 1 Department of Pharmacology, Kathmandu Medical College Teaching Hospital, Duwakot, Bhaktapur, Nepal; 2 Department of Community Medicine, Kathmandu Medical College Teaching Hospital, Duwakot, Bhaktapur, Nepal; 3 Department of General Surgery, Montefiore Medical Center, Bronx, New York, USA

**Keywords:** *COVID-19*, *Nepal*, *self-medication*

## Abstract

**Introduction:**

Self-medication is a common practice worldwide. Major problems related to selfmedication are wastage of resources, increased resistance of pathogens, adverse reactions, and prolonged suffering. This study aimed to find the prevalence of self-medication among medical students and staffs of a tertiary care centre during the COVID-19 pandemic.

**Methods:**

A descriptive cross-sectional study was conducted among medical students and staffs of a tertiary care centre from 1^st^ November to 30^th^ November, 2021. Ethical clearance was taken from the Institutional Review Committee (Reference number: 2710202102). Convenience sampling was done to reach the sample size. Online questionnaires consisting of information on self-medication and socio-demographic characteristics were used. The data was transferred into an Excel spreadsheet and later was exported to Statistical Package for the Social Sciences version 20 for analysis. Point estimate at 95% confidence interval was calculated along with frequency and proportion for binary data.

**Results:**

Among 383 participants, the prevalence of self-medication during the pandemic was 193 (50.4%) (45.39-55.40 at 95% Confidence Interval). About half of the respondents 90 (50.3%) who selfmedicated purchased the medicines directly from the pharmacy. The most consumed medicines were Paracetamol 128 (18.9%), Vitamin C 126 (18.6%), Zinc 86 (12.7%), Multivitamins 75 (11.1%), and Vitamin D 65 (9.6%) followed by Azithromycin 54 (8%), cough syrup 53 (7.8%) and Ibuprofen 46 (6.8%).

**Conclusions:**

The prevalence of self-medication during the COVID-19 pandemic is lower compared to that of other developing countries. Paracetamol and Vitamin C are the most consumed drugs for self-medication and Azithromycin is the most used prescription-only drug for self-medication during the COVID-19 pandemic.

## INTRODUCTION

The World Health Organization defines self-medication as the selection and utilization of medicines to treat selfrecognized symptoms or ailments without consulting a physician.^1^ Family, friends, neighbours, the pharmacist, previous prescribed drug, or suggestions from an advertisement in newspapers or popular magazines are common sources of self-medication.^2^

Self-medication is a common practice worldwide.^3^ During the pandemic, the constant fear of going outside and using health services may have an impact on the use of self-medication.^4^ At this stage of the pandemic, self-medication is found as a common practice in many countries.^5^ Major problems related to self-medication are wastage of resources, increased resistance of pathogens, and serious health hazards such as adverse reaction and prolonged suffering. Antimicrobial resistance is a current problem worldwide particularly in developing countries where antibiotics are available without any prescription.^6^

This study aimed to find the prevalence of selfmedication among medical students and staffs of a tertiary care centre of Nepal during the COVID-19 pandemic.

## METHODS

This was a descriptive cross-sectional study conducted from 1^st^ November to 30^th^ November, 2021. Ethical approval was taken from Institutional Review Committee (IRC) Kathmandu Medical College Teaching Hospital (Reference No: 2710202102). Consent was attached at the beginning of the Google form, those who agreed could proceed further. Medical students and staffs, affiliated with Kathmandu Medical College Teaching Hospital (KMCTH) were included and the incomplete data forms were excluded. A convenience sampling method was used.

The sample size was calculated by using the formula:

n = Z^2^ × (p × q) / e^2^

  = (1.96)^2^ × 0.50 × (1-0.50) / (0.06)^2^

  = 267

Where,

n= required sample size
Z= 1.96 at 95% of Confidence Interval (CI)
p= prevalence of self-medication among medical students and staffs of a tertiary care centre taken as 50% for maximum sample size
q= 1-p
e= margin of error, 6%

The minimum sample size calculated was 267. Taking 10% non-response rate, the sample size becomes 294. However, the total sample size taken was 383.

The main instrument to collect data was an online questionnaire. An online questionnaire was prepared using Google forms and was sent to participants through email, social media (Facebook, Instagram, Viber, and Whatsapp). The survey was kept brief to achieve greater response rates. Pretest was conducted among 20 participants for clarity and content prior to dissemination for others. Day-to-day supervision of sent questionnaires was done. Those who did not respond were sent up to three reminders.

The data was transferred into an Excel spreadsheet and later exported to Statistical Package for the Social Sciences version 20 for data analysis. Descriptive statistics were used, and variables were represented in terms of percentages. Point estimate at 95% confidence interval was calculated along with frequency and proportion for binary data.

## RESULTS

Among 383 participants, the prevalence of selfmedication during the pandemic was 193 (50.4%) (45.39-55.40 at 95% Confidence Interval). About half of the respondents 90 (50.3%) who self-medicated purchased the medicines directly from the pharmacy. Among the respondents who participated in the survey of which 237 (61.9%) were from the Kathmandu Valley. Among all, 194 (50.7%) were between 18-24 years of age. All the respondents were literate, and 291 (76 %) had attended University. The number of male and female respondents was similar, with females being 194 (50.7%). Majority of the respondents were unmarried 235 (61.4%). Around 196 (51.2%) participants were students whereas 93 (24.3%) were in health-care related careers (Table 1).

**Table 1:** Socio-demographic characteristics.

Characteristics	n (%)
**Age**	
18-24	194 (50.7)
25-39	155 (40.5)
40-59	32 (8.3)
60-65	2 (0.5)
**Sex**	
Female	194 (50.7)
Male	189 (49.3)
**Marital status**	
Single	235 (61.4)
Married	146 (38.1)
Divorced	2 (0.5)
**Residence**	
Kathmandu Valley	237 (61.9)
Outside Kathmandu Valley	146 (38.1)
**Highest level of education**	
High School	78 (20.4)
Diploma	14 (3.6)
University or College	291 (76)
**Employment status**	
Student	196 (51.2)
Health-care related career	93 (24.3)
Employee	77 (20.1)
Business	17 (4.4)

About 106 (27.7%) respondents reported that they had been or were currently infected with COVID-19. About 58 (15.1%) respondents were suffering from co-morbidities like Hypertension 21 (36.2%), Thyroid issues 24 (41.4%), Diabetes 5 (8.6%) and others 8 (13.8%).

The respondents who practised self-medication were 193 (50.4%). Among them, 84 (21.9%) self-medicated when symptoms of COVID-19 developed. About 90 (50.3%) respondents who self-medicated purchased the medicines directly from the pharmacy (Table 2).

**Table 2:** Use of self-medication.

Variable		n (%)
Self-medication	Yes	193 (50.4)
	No	190 (49.6)
When did self-medication start	When confirmed PCR test positive	43 (11.2)
	When symptoms of COVID-19 developed	84 (21.9)
	Without symptom for prevention	66 (17.2)
Source of medicine	Prescription written for family	26 (14.5)
	Advice from friend	41 (22.9)
	Directly requested from pharmacy	90 (50.3)
	Tele-medicine	22 (12.3)

Common symptoms during the pandemic for which the respondents took medications were fever 116 (26.1%), body ache 92 (20.7%), throat pain 76 (17.1%), dry cough 74 (16.6%), loss of smell/taste 68 (15.3%) and diarrhoea 19 (4.3%).

The most consumed medicines were Paracetamol 128 (18.9), Vitamin C 126 (18.6%), Zinc 86 (12.7%), Multivitamins 75 (11.1%) and Vitamin D 65 (9.6%) followed by Azithromycin 54 (8%), cough syrup 53 (7.8%) and Ibuprofen 46 (6.8%) (Table 3).

**Table 3:** List of medicines (Multiple responses).

Medicine	n (%)
Paracetamol	128 (18.9)
Ibuprofen	46 (6.8)
Azithromycin	54 (8)
Chloroquine	2 (0.3)
Vitamin C	126 (18.6)
Vitamin D	65 (9.6)
Zinc	86 (12.7)
Multivitamins	75 (11.1)
Dexamethasone	4 (0.6)
Ivermectin	8 (1.2)
Montelukast	5 (0.7)
Calcium	22 (3.2)
Cough Syrup	53 (7.8)
Other	3 (0.4)

A total of 42 (11%) respondents faced adverse effects from the use of these drugs. Some common adverse effects encountered were headache 21 (26.2%), allergic reactions 16 (20.1%), dizziness 15 (18.8), gastritis 12 (15%), constipation 8 (10%), diarrhoea 5 (6.2%) and fungal infection 3 (3.8%).

## DISCUSSION

The magnitude of self-medication during the COVID-19 pandemic in our study was 193 (50.4%). Three studies in Pakistan, Bangladesh, and Togo have reported the prevalence of self-medication during the COVID-19 pandemic to be 53%, 71.40 %, and 34.2% respectively.^7-9^ The prevalence of self-medication in Pakistan was similar to this study whereas it was much higher in Bangladesh and lower in Togo.

In this study, 17.2% of respondents medicated without any symptoms for prevention. This was consistent with the study in Bangladesh.^8^ The majority in our study, who selfmedicated, obtained the medicines directly from a pharmacy. The study in Pakistan mentioned, for the majority, the source of the drug was either a prescription written for a family member or directly from the pharmacy.^7^ Directly from the pharmacy may be a common source of self-medication in countries like Nepal and Pakistan because dispensing of medicines from pharmacies is not strictly regulated in these regions.

The medicines most commonly used in our study were Paracetamol, Vitamin C, and Zinc. Drugs like Ivermectin, Montelukast, Dexamethasone, and Chloroquine were used by 0.2%-1.3% of respondents. Various studies have shown drugs like Ivermectin, Azithromycin, Doxycycline, Hydrochloroquinone as the topmost consumed drugs.^7,8^ While; two studies in Peru and Togo have Acetaminophen (paracetamol) and Vitamin C and as the most consumed medicine for self-medication respectively.^5,9^

The most used 'prescription-only' drug for selfmedication was Azithromycin. Though the use of Azithromycin has increased during the COVID-19 pandemic, routine use of azithromycin for COVID-19 in absence of additional indication is not recommended.^10,11^ Instead, inappropriate use of antibiotics can further lead to increased antimicrobial resistance. Adverse effects were seen using medicines which were also seen in other studies.^7,12^

The limitation of this study was that the limited sample size and the convenience sampling technique could not make the results representative of the entire population.

## CONCLUSIONS

This study concludes that the prevalence of selfmedication during the COVID-19 pandemic was lower compared to that of other developing countries. Paracetamol and Vitamin C were the most consumed drugs for self-medication and Azithromycin was the most used prescription-only drug for self-medication during the COVID-19 pandemic.
